# How parts make up wholes

**DOI:** 10.3389/fphys.2012.00455

**Published:** 2012-11-30

**Authors:** Scott D. Findlay, Paul Thagard

**Affiliations:** ^1^Department of Anatomy and Cell Biology, Western UniversityLondon, ON, Canada; ^2^Department of Philosophy, University of WaterlooWaterloo, ON, Canada

**Keywords:** parts, wholes, organization, constitution, hierarchies, levels, emergence, family

## Abstract

We propose a schema that characterizes how parts constitute wholes at diverse levels of organization, ranging from the atomic to the biological to the social. This schema of tags, organizers, attachers, and communicators provides a unified understanding of the structure, function, and dynamics of organization in physics, biology, and the cognitive and social sciences. We use this schema to identify and describe structures and processes at many levels of organization, and discuss its relevance for understanding the nature of constitution and emergence, especially the relation between individual humans and the social groups they constitute.

## Introduction

Part-whole relations are important in all sciences, ranging from physics, where atoms are composed of electrons and other particles, to sociology, where groups are composed of individual people. Relations of constitution are especially central to biological theories that investigate how molecules make up cells, how cells make up organs, how organs make up organisms, and how organisms make up populations. Many biologists have stressed the importance of hierarchies of organization in living systems (e.g., Woodger, 1929; Novikoff, 1945; MacMahon et al., 1978; Miller, 1978; Mayr, 1982; Eldredge, 1985; Zylstra, 1992; Heylighen, 2000; McShea, 2001; Valentine, 2003; Korn, 2005; Lane, 2006; Pavé, 2006). Similarly, many cognitive scientists have considered thinking at biological and social levels as well as psychological ones (e.g., Simon, 1962, 1996; Newell, 1990; Churchland and Sejnowski, 1992; Holland, 1998; Thagard, 2006). Philosophers of science have discussed how the organization of entities at multiple levels can be relevant to explaining biological and cognitive operations (e.g., Bechtel and Richardson, 1993; Thagard, 1999, 2010; McCauley and Bechtel, 2001; Bunge, 2003; Darden, 2006; Craver, 2007; Craver and Bechtel, 2007; Wimsatt, 2007; Bechtel, 2008; McCauley, 2009; Winther, 2011). Nevertheless, the nature of part-whole relations has largely been taken for granted, with little systematic attempt to say how components at a lower level of organization constitute wholes at a higher level.

We propose a general schema that characterizes how parts make up wholes at all levels of organization. To put it concisely, parts have identifying tags that allow organizers, attachers, and communicators to make the parts work together as a whole. Constitution is not a static relation between parts and wholes, but rather a combination of processes in which the parts interact to compose and maintain the whole. Such dynamics help to explain the relationship between individual people and social groups such as families.

## A part-whole schema

We conjecture that part-whole relations at all levels arise from the following factors:

1. *Parts* are the units that assemble together to form a whole.
2. *Tags* are properties of parts that give structural and/or functional identities.
3. *Organizers* are forces or processes that bring parts together into structural and/or functional relationships.
4. *Attachers* are forces, processes, or entities that hold parts together.
5. *Communicators* are specialized components that move to allow interactions among physically separated parts.
6. *Wholes* are structures made of parts that together operate as a system; wholes can also function as parts in higher-level wholes.

**Figure 1:**
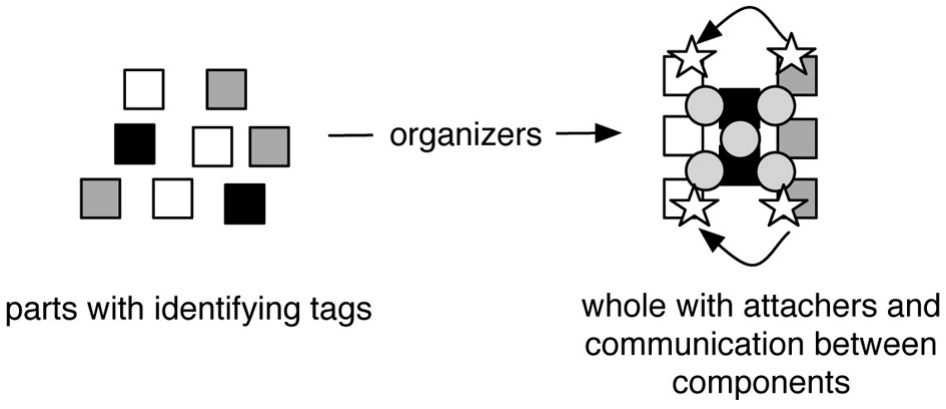
**Organization at a given level.** Squares represent components with different tags represented by different shades. The organizers arrow represents ongoing processes that pattern the structural and functional arrangement of parts into a whole. Circles represent attachers. Stars represent communicators that move between parts as indicated by curved arrows.

In this schema, parts have identities based on their tags that determine how they are brought together into specific arrangements by organizers. Parts are held together by attachers, and the operation of wholes is also influenced by communication between physically separated parts. Figure 1 illustrates how tags, organizers, attachers, and communicators, enable parts to constitute a whole.

The process of organization can be understood by analogy to the familiar human practice of construction. Composing biological wholes like molecules and cells is roughly like building an item of furniture such as a table. Building a table (the whole) from pieces of wood (the parts) requires tags, organizers, and attachers. Each piece of wood has tags based on its dimensions and type, for example, a 2-by-4 piece of cherry. Organizers that shape and arrange the pieces include processes such as sawing, hammering, and clamping. Finally, the attachers are nails and glues that hold the table together. Organizers differ from attachers in that the former are forces or processes that bring parts together, whereas the latter are forces, processes, or things that hold parts together in a continuing pattern. Builders use organizers like hammering to bring parts together, and attachers like nails to make them stay together.

Two key differences exist between biological systems and artifacts like tables. First, biological wholes are rarely assembled or made functional by the laying out of individual parts and subsequent stepwise assembly of these parts. Instead, some wholes such as tissues, organs, and organ systems develop together, while other wholes such as atoms do not need to be assembled within an organism. Second, while many pieces of furniture and appliances manufactured by humans are static wholes, all levels of biological organization require ongoing and dynamic interactions to both establish and maintain the structure and function of biological entities. Unlike artifacts, biological systems are self-organizing and self-maintaining. Dynamics at a given level govern the replacement of components over time and their movement within a whole. While components of a structure like a table rarely need to be replaced, the biological world consists of wholes with parts that are continuously being replenished: new parts are synthesized, tagged, organized, and attached to maintain the structural and functional integrity of the whole.

## Levels of organization

**Figure 2:**
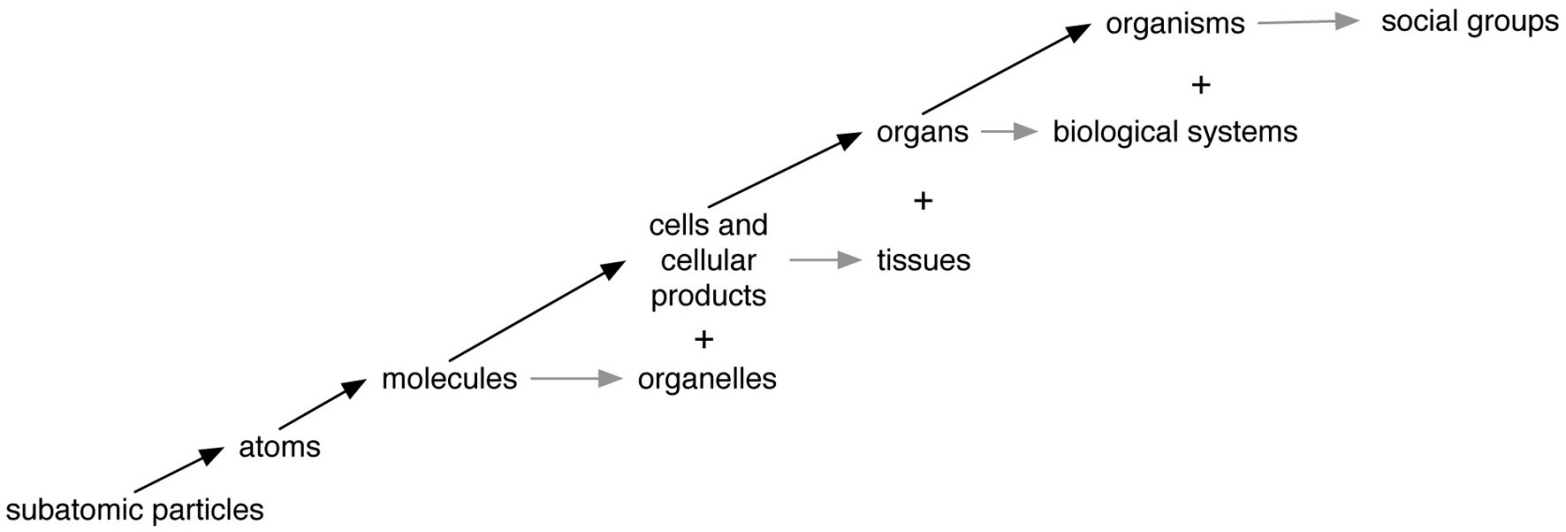
**The levels of organization most relevant to humans.** Moving up or to the right represents a higher level of organization. Arrows represent ongoing dynamics that establish and/or maintain structure and function. Black arrows represent relations between primarily structural parts and wholes, while gray arrows represent primarily functional collections of parts within a structural whole. The “+” sign indicates that lower level entities can be grouped into higher level ones in ways different from the basic part-whole relation.

We will now show how the part-whole schema applies at many different levels of organization. Many biologists, cognitive scientists, and philosophers have offered accounts of relevant levels, including one author who proposes that life includes 19 different subsystems (Miller, 1978). Figure 2 depicts some of the levels that we think are most needed for explaining important phenomena. Much more detailed accounts of the parts and wholes at each level will be given below.

Table 1 telegraphically summarizes how the part-whole schema applies at each of the levels in Figure 2. Details about the relevant tags, organizers, attachers, and communicators are given in the following discussion of how relations of constitution connect levels of organization. A fuller account of the processes that make parts into wholes would need to specify the environments in which they operate including the interactions between external entities and internal parts. In addition to the dynamism of part-whole relations discussed here, there are also changes at multiple levels of organization across evolutionary time. Indeed, evolution is driven by variation and the natural selection of beneficial traits that collectively define a complex organism. Note that some examples in Table 1 apply only to the main structural whole (e.g., cells) and not functional sub-level wholes (e.g., organelles). Table 1 provides typical examples of part-whole relations, not an exhaustive list.

**Table 1:** **Examples of major tags, organizers, attachers, and communicators for various levels of organization**.

**Parts**	**Whole**	**Tags**	**Organizers**	**Attachers**	**Communicators**
Subatomic particles (protons, neutrons, electrons)	Atom	• Physical properties (e.g., mass and charge)	• The four fundamental forces (strong nuclear, electromagnetism, weak nuclear, gravity)	• Field carriers of the fundamental forces (gluons, photons, weak gauge bosons, gravitons)	• Quantum entanglement
Atoms	Molecule	Electron density Proximity to other atoms	Brownian motion Chemical reactions	Covalent bonds Hydrogen bonds van der Waals interactions Hydrophobic effects (e.g., in protein folding)	• None
Molecules	Organelle/cell	Physical and chemical properties Sequence identity Localization sequences Post-translational modifications	Energetically and physically favorable interactions Intracellular transport Chemical reactions (signal transduction, metabolism) Cell division	Attractive forces between components Membranes The cytosol The cytoskeleton	Proteins/enzymes Electrons Ions Small molecules (e.g., ATP) mRNA
Cells and cellular products	Tissue/organ	Location Cell surface Molecules Differentiation state (e.g., polarity, secretions)	Cellular processes (e.g., migration, polarization, differentiation) Cell signaling Developmental processes (e.g., organogenesis)	Interlocking membranes Junctions Extracellular matrix components Connective tissue	Motile cells Ions Small molecules (e.g., growth factors, neurotransmit-ters)
Organs	Organ system/individual organism	Embryonic origin (ectoderm, mesoderm, endoderm) Location Connections Special abilities	Developmental programs Movement of substances through networks (e.g., blood flow)	Cavities Connective tissues Membranes	Small molecules (e.g., hormones) Specialized connective tissues (blood and lymph)
Individual organisms	Social group	Physical features Behavioral characteristics Mental representation	Social events (e.g., parties, meetings, rituals) Institutions and organizations (e.g., universities, corporations) Cognitive processes (e.g., planning)	Shared environments (e.g., houses, churches) Emotional mental states Social practices	Sounds Gestures Speech Media

Different presentations of the somatic hierarchy vary in what levels are included. Here we classify entities as either “main levels” or “sub-levels” based on two distinct classes of relationship between entities. The class of relationship depends on three factors: exclusivity of composition, autonomy, and structural/functional nature. Importantly, the classification of an entity as a main level or sub-level is not meant to be rigid, but is simply a best fit based on these three factors and involves some degree of arbitrariness (Zylstra, 1992).

First, we take as a main level the lowest level entity that a whole must be broken down into to account for its entire structure. For example, cells are not made entirely of organelles, but are made entirely of molecules. Therefore molecules and cells are main levels, and organelles are a sub-level. Second, the main levels of this organization are entities related by “whole–whole” relations (Zylstra, 1992). These entities have a relative degree of autonomy and are a distinct unit. The sub levels of this organization represent “part-whole” relationships. These entities have an identity only within the context of the whole they make up. To borrow an analogy from Zylstra, a whole–whole relation relates furniture such as chairs and tables to the house that they are found in. Tables are functional wholes themselves, and it is easy to imagine a table being functional in the absence of a house, yet tables are still key functional components of houses. Conversely, part-whole relationships describe how walls and floors of the house relate to the house that they combine together to form. The walls and floors of a house do not have much utility or autonomy independent of the structure that they collectively constitute.

Third, main levels are entities that take on a well-defined structure, while sub-levels are often functional collections of lower level entities with a dispersed or irregular structure. For example, a cell (a main level) has a well-defined structure defined by a membrane that encapsulates its contents. However, organelles (a sub-level) such as the Golgi apparatus exist as a network of bodies that do not take on a well-defined structure within the cell.

Our part-whole schema of tags, organizers, attachers, and communicators is intended to be ontological as well as conceptual and epistemological. All levels of organization require ongoing dynamic interactions to establish and maintain structure and function. Biological entities often display structure-function duality. For example, cells are a main structural unit of organisms in constituting bones, muscles, and connective tissue, but are also responsible for carrying out physiological tasks such as metabolism, protein production, and waste disposal. Thus it is important that the assembly schema in Figure 1 represents both structural and functional components of biological organization and the integral relationship between these two realms, as advocated by Zylstra (1992). A component such as a protein molecule can be a part of a higher level structure such as a cell, but that same protein molecule can also double as a functional component of a higher level mechanism such as cell metabolism. Similarly, components can be tagged to determine how they will fit together structurally, or which other components they will interact with in a mechanism. Organizers bring both structural and functional components into higher level structures and/or mechanisms.

## Applications of the part-whole schema

Let us see in more detail how these principles apply to particular cases of constitution.

### Subatomic particles into atoms

We start at the level of subatomic particles, where electrons, protons, and neutrons and other components are arranged into whole atoms (Martin, 2009). At this level the tags are the physical properties of the particles such as mass and charge. The organizers are the four fundamental forces that explain the interactions of subatomic particles based on the mass and charge that tag them. These forces are, in order of decreasing relative strength, the strong nuclear force, electromagnetism, the weak nuclear force, and gravity. These organizers are physically manifested in field carriers that mediate each fundamental force; the strong nuclear force by gluons, electromagnetism by photons, the weak nuclear force by the weak gauge bosons, and gravity by gravitons, although the existence of gravitons has not yet been experimentally confirmed. Therefore it makes sense to think of these field carriers as the physical attachers holding electrons, protons, and neutrons into atoms. The graviton is the only attacher that manifests an exclusively attractive force.

The dynamics of subatomic particles can be quite complicated, but some simple instances can be mentioned. Electrons are not present in static positions or even regular orbits around nuclei. Additionally, the subatomic organizers and attachers must be continuously active to maintain the structure and function of atoms. Hence the constitution of atoms by subatomic particles is a highly dynamic process.

### Atoms into molecules

Atoms are parts that are tagged with an electron density or net charge that dictates their tendency to undergo chemical reactions that group atoms into molecules. The organizers at this level include the random movement of atoms known as Brownian motion, the sharing of electrons between atoms of different molecules in energetically favorable ways, and enzymes that enhance the rate of many biologically relevant chemical reactions. Chemical reactions generate attachers in the form of chemical bonds that form between atoms. Covalent bonds are relatively very strong and more permanent; hydrogen bonds are individually relatively weak and transient, but collectively strong and more permanent; and van der Waals interactions are individually very weak, but of significant strength for large molecules (Alberts et al., 2004). Although collections of individual atoms are rarely laid out and systematically assembled into large biomolecules, the same tags, organizers, and attachers can explain the formation of large macromolecules, often from smaller molecular components. Indeed, some of the most important biomolecules such as nucleic acids and proteins consist of long polymer chains of smaller monomer molecules. Importantly, this structure helps define the function of these molecules. For example, secondary and tertiary protein structure is heavily influenced by the order and properties of the amino acid subunits that make up a protein. Similarly, the genetic code is defined by the sequence of nucleotide subunits (A, T, G, and C) that make up a DNA molecule.

The relations between atoms in molecules are dynamic given that free atoms as well as those in molecules always exhibit some form of motion, whether vibrational, rotational, or translational. Additionally, the electron components of atoms that participate in chemical bonds are far from static as discussed above. Therefore, the part-whole relations in molecules are highly dynamic in nature. The molecular reactants and products of biochemical reactions exist in equilibrium, so the reaction continues but reaches a state where the forward and reverse reactions proceed at the same rate.

### Molecules into organelles and cells

Many molecules within the cell are grouped into discrete functional compartments known as organelles. While the most familiar of these structures include the membrane-bound nucleus, endoplasmic reticulum, and mitochondria (Alberts et al., 2004), some texts also recognize large molecules within the cell as non-membranous organelles (Martini et al., 2006). Organelles represent a sub-level of biological organization since cells are also composed of molecules that constitute both the intracellular space between organelles known as the cytosol, as well as the cell membrane.

The arrangement of molecules into both organelles and entire cells involves a variety of tags. Often simple chemical properties of molecular components such as electrostatic charge or the ability to form hydrogen bonds with other molecules serve as tags for organization (Alberts et al., 2004). In other cases such as protein interactions, additional tags such as three dimensional shape determine the tolerated array of structural and functional relationships. More amazingly, protein molecules can contain sorting signals within their amino acid sequence that direct them to a specific organelle within the cell. Additional molecular tags include modifications to a protein after it has been synthesized or translated from the genetic code, known as post-translational modifications. These modifications involve the addition of different chemical groups by different enzymes that quite literally *tag* the protein. Phosphorylation is the main modification used in intracellular signaling cascades and is often used to activate or deactivate a given enzyme (Voet et al., 2008). Another widespread modification is the acetylation of histones that diminishes the attractive force between these proteins and DNA, allowing increased gene expression (Alberts et al., 2004).

The arrangement of molecules into functional organelles and cells depends on a number of organizers. Organization involves cooperation between passive principles such as energetically and physically favorable arrangements of molecular components, and more active cellular processes. The cell membrane is an example of this complementarity. The phospholipid components of cell membranes spontaneously form a bilayer due to the amphipathic nature of phospholipids that have a hydrophobic tail and a hydrophilic head region. However, active processes such as biosynthetic chemical reactions and cell division are also required to build and maintain cell membranes. Major cellular processes such as metabolism, signal transduction, and intracellular transport are all important organizers.

To physically hold tagged molecular components together into organelles and cells, nature needs attachers. Some molecules are able to associate based primarily on their own properties and passive organizers as discussed above. However, there are two other attachers that are found throughout the cell. The cytosol is an attacher since it is packed with an abundance of molecules that give it a gel-like consistency and support embedded organelles. Another widespread attacher is the cytoskeleton that is made up of actin, intermediate filaments, and microtubules, that help maintain the shape and structural integrity of the cell.

Communicators within organelles and cells are involved in the functionality of these entities by moving to mediate interactions between physically separate parts. For example, enzymes in signaling pathways mediate the effects of an extracellular growth factor on gene transcription in the nucleus, even though the particular growth factor and gene never have a direct physical interaction. In mitochondria, the movement of protons (H^+^) across the inner membrane connects events of the electron transport chain with the generation ATP, the cell's energy currency. Other communicators such as messenger RNA also allow genetic programs stored as DNA within the nucleus to affect cellular processes throughout the cell by directing protein synthesis.

From the examples of organizers above, it is quite obvious that the relationship between molecules and the cell is highly dynamic. Additionally, since many molecules such as proteins usually have a finite lifespan, even terminally differentiated cells need to remain metabolically active to maintain their structural and functional integrity. Along with being regenerated, such molecules must be actively tagged, organized, attached, and moved if they are to be communicators. Many molecular components are also in constant motion within the cell. This motion can take the form of passive diffusion through the cytoplasm or an organelle complex, or it can be directed by active intracellular transport. In the latter case, microtubules that are polymers of a protein known as tubulin, along with motor proteins, provide a transportation infrastructure within the cell, along which organelles can be moved. Such movement is important in processes such as the establishment of cell polarity, as when a neuron develops an axon at one end and dendrites at the other. Not surprisingly, the microtubule network itself is highly dynamic, adjusting rates of assembly and disassembly in what is known as “dynamic instability” to facilitate the desired transportation.

### Cells and cell products into tissues and organs

A tissue is a collection of cells and cell products that function together to carry out a limited and specific function. The four primary tissue types are epithelia, connective tissues, muscle tissue, and neural tissue (Martini et al., 2006). Two or more tissues combine to form an organ with a more complex vital function. Tissues represent a sub-level of biological organization since the functionality of organs relies upon individual cells such as macrophages, neurons, and lymphocytes in addition to component tissues (Alberts et al., 2004).

Cell tags come in the form of molecules expressed on the surface of cells. Expression of different types of cell surface molecules known as cell adhesion molecules determines which cells are able to stick together. Other cell surface molecules are functional tags that determine the functional role of a cell within the context of an organ. For example, B cells of the immune system are able to proliferate and produce antibodies in response to a pathogen due to expression of a specific B cell surface receptor (Murphy et al., 2008). Other tags that determine how a cell will function within a tissue or organ include aspects of its differentiation manifested by features such as polarity and type of protein secretions.

Organizers include a variety of molecular processes both within and between cells. A specific example of a protein that is central to the organization of cells into tissues is a member of the cadherin family of cell adhesion molecules known as Fat. Fat prevents cells from becoming too large, and guides planar cell polarity within a tissue such as epithelia (Sopko and McNeill, 2009). For organs, recent research suggests that different “organ identity genes” might direct the development of organs as biological wholes, as has been shown for the gene Pha-4 for pharynx development in the worm (Mango, 2009).

Several classes of junctions composed of clusters of cell adhesion molecules, adaptor proteins, and branches of the cytoskeleton serve as attachers that physically hold cells together (Alberts et al., 2004). Other attachers include components of the extracellular matrix deposited by cells, such as proteoglycans that have even been termed “intercellular cement,” more complex connective tissue, and the simple physical interlocking of membranes as occurs in some types of epithelia (Martini et al., 2006).

Communicators are any entities that move between components within a tissue or organ. These include migratory cells such as many cells of the immune system (Murphy et al., 2008), as well as molecules and ions that allow intercellular communication within a tissue or organ, for example, neurotransmitters between neurons in the brain.

The relationship between cells and tissues or organs involves ongoing, dynamic interactions. We have already discussed interactions involving intercellular communication within tissues and cell movement within organs. For tissue maintenance, the cellular components of tissues classified as renewal such as the epidermis of the skin contain a proliferative zone of stem cells that continuously replace a population of short-lived differentiated cells (Slack, 2006). Even tissues classified as post-mitotic and expanding need to be replenished to certain extents. As with molecules, new cells need to be tagged, organized, attached, and moved if they are communicators, in order to contribute to functional tissues and organs. Furthermore, in order to survive and remain functional, cells of established tissue must continually receive signals from their environment and respond appropriately, express cell adhesion molecules to maintain appropriate structural connections, and maintain patterns of gene expression to allow retention of cellular identity by progeny cells.

Human brains consist of billions of cells, including neurons and glial cells that provide nutrients and serve as attachers by holding neurons in place. Among the most important kinds of tags that neurons possess is their synaptic connections that enable them to communicate with other neurons. Interconnections among large numbers of neurons support high level mental operations including both cognition and emotion (Schröder and Thagard, in press; Thagard, 2010; Thagard and Schröder, forthcoming). Neural functioning also enables brains to communicate with other organs such as the heart and limbs.

### Organs into organ systems and individuals

Organs are formed into functional biological systems that work together to collectively constitute a living individual organism. Clusters of cells in the early embryo are tagged based on their early commitments as one of the three primary germ layers (endoderm, mesoderm, and ectoderm). These very early tags influence which cells will be grouped into a given biological system, and the arrangement of organs within the body. Once humans are fully developed, tags that determine an organ's functionality include the location that it is found within the body, its associations and connections with other organs, and any specialized abilities. For example, the heart's functional tags include its location within the chest, connection to major arteries and veins, and ability to pump blood due to the contraction of cardiac muscle tissue and the coordinated activities of its other tissue components.

Organizers that arrange organs into an organism are the complex processes of growth and development. For example, as the diaphragm develops it separates organs of the thoracic cavity from those of the abdominopelvic cavity (Martini et al., 2006). Evolutionary constraints on development contribute to what is known as the vertebrate body plan and explain common features and morphology of vertebrates (Slack, 2006). The body plan is believed to represent a real feature of organization as evidenced by the highly conserved *Hox* genes. These genes encode transcription factors important in body patterning during various developmental stages (Voet et al., 2008). The functional organizers of biological systems are processes that connect parts in functional relationships such as angiogenesis (the formation of new blood vessels). Such organizers allow the flow of communicators such as blood and lymph (both specialized connective tissues) and hormones that can cause effects across organs in a biological system or throughout the body. Structural attachers that maintain organs in their proper arrangements within an organism include body cavities that house organs, connective tissues that support them, and membranes that line them (Martini et al., 2006).

Dynamics involve restricted movement of organs such as the beating of the heart or swelling of the fed stomach. The internal body cavities provide a low friction environment for organs to expand or move into. An example is the pericardial cavity that supports the beating heart, making slippery contacts with the heart and the surrounding tissues. At the lower levels discussed so far, components are recycled over relatively short periods of time. However, organs such as the heart and liver do not normally spontaneously cease to function or exist as wholes much like most molecules and cells do at some point in time. Nor are they instantaneously replaced by substitute organs waiting in the wings. Instead entire organs are replenished gradually by the renewal of lower level components such as cells and molecules.

### Individual organisms into social groups

Our schema for part-whole relations also applies to social groups, which consist of collections of individual organisms. Non-human animals form groups such as herds, packs, flocks, and schools of fish. Human individuals collect into functional social groups such as companies, families, teams, congregations, and circles of friends. For humans, the tags include physical features, behavioral characteristics, contextual cues and mental representations that help people identify other individuals as relatives, friends, or coworkers. The principal organizers involved include brain processes relevant to planning, such as memories, emotions, values, and goals. We need these cognitive processes to plan, comprehend, judge, and understand our interactions with others and arrange ourselves in functional groups that allow us to meet our goals as social creatures. Other organizers are events and processes such as family dinners, parties, and religious rituals. The attachers in social groups are primarily emotional representations that people have of each other that establish social bonds, for example in couples, families, and teams (Thagard, 2012, in press). Other attachers include places and events that hold people together in social groups, such as houses, office buildings, and places of worship.

Communicators at the social level include sound waves that convey words from one person to another, as well as modern devices such as telephones and computers. As shown in Figure 2, the relation between individuals and social groups is primarily functional, like the relation between organs and biological systems such as the respiratory system, not mainly structural like the relation between cells and organs. Nevertheless, the part-whole schema still applies, as the functions of social groups arise from the tags, organizers, attachers, and communicators that make the individuals into a group.

Social groups obviously involve complex dynamics. New members can join a group and old ones can leave. Individuals within a group can also move physically, interacting with different individuals of the group over the course of a social gathering. An individual's relationship relative to others within the group can also change. For example, an individual with the relation *mother* in a family can add *grandmother* to her identity upon her daughter having a child, or an assistant within a corporation can be promoted to executive. Moreover, these dynamics depend on the extraordinarily complex brain processes that mediate individuals' evolving relationships with others. For example, memories of others can be formed, remembered correctly or incorrectly, and even forgotten entirely. Over the span of a century, virtually none of the individual members of a given social group stay the same, yet the traditions and rituals that define social groups such as families, religious groups, and corporations can remain strong.

## Constitution and emergence

The application of our schema to six different levels of organization supports its plausibility as a general account of part-whole relations. We now use it to address important problems concerning the nature of constitution and emergence. The part-whole schema provides an understanding of emergence that is an alternative both to reductionism, according to which wholes can be fully explained in terms of their parts, and to holism, according to which wholes can be understood without considering the operations of their parts.

Constitution of parts into wholes requires the whole complex of tags, organizers, attachers, and communicators. The tags of the parts allow them to be organized by forces and processes and attached together into wholes in which communicators provide additional interactions among parts. As a result, the wholes can have emergent properties, which are properties that belong to the wholes, do not belong to any of the parts, and are not aggregates of properties of the parts (cf. Bunge, 2003; Wimsatt, 2007). In contrast, an aggregate property is a simple sum: for example, the weight of a table is just the sum of the weights of the pieces of the table. Emergent properties differ from aggregates because they arise from three kinds of interactions among parts: organizing, attaching, and communicating. Constitution is a relation that results from many mechanisms responsible for organizing, attaching, and communication. Table 2 provides examples of constitution and emergence at six levels, and the next section goes into more detail concerning how individuals make up social groups.

**Table 2:** **Examples of constitution and emergence**.

**Parts**	**Whole**	**Constitution**	**Emergent properties**
Subatomic particles	Atom	The mass and charge of particles allow them to be organized by fundamental forces and attached by field carriers into whole atoms	Atoms are capable of bonding into molecules
Atoms	Molecule	The electron densities of atoms allow them to be organized by chemical reactions and attached by covalent bonds into whole molecules	Molecules may be different states of matter (gas, liquid, solid) from their collections of atoms
Molecules	Organelle/cell	The chemical properties of the molecules allow them be organized by chemical reactions and attached by the cytoskeleton into whole cells in which proteins and ions provide communication	Cells are able to survive by obtaining energy and to reproduce by division
Cells and cellular products	Tissue/organ	The locations and surface molecules of cells allow them to be organized by processes such as migration and attached by junctions into whole tissues or organs in which small molecules provide communication	Organs are able to accomplish complex biological functions such as pumping blood
Organs	Organ system/individual organism	Organs develop in specific locations within the body where attachers such as connective tissue and communicators such as hormones allow them to function as organ systems within an organism	Organ systems are able to accomplish even more complex biological functions such as providing nutrients to a whole organism
Individual organisms	Social group	The physical and behavioral properties of organisms allow them to be organized by events and institutions and attached by environments and mental states into social wholes in which sounds and other signals provide communication	Social groups are capable of collective actions such as animal swarming, human politics, and economic markets

The examples given are illustrative, not comprehensive: at each level there are other cases of constitution and emergence than the ones presented in the table.

## Families and the people/group problem

One of the key problems in social science is the relation between individual people and the social groups to which they belong, which we will call the *people/group* problem, although it could just as well be called the psychological/social problem or the agent/structure problem. It can be stated in different ways, including:

1. What is the relation between individual agents and social structures such as institutions and states (see e.g., Giddens, 1984; Giddens, Wendt, 1999).
2. What is the relation between the psychology of individual humans (which contemporary cognitive science understands in terms of mental representations and processes) and the investigation of social changes carried out in fields such as sociology, anthropology, economics, and political science?

The two most prominent and extreme accounts of the relation between the social and the psychological are the methodological individualist, reductionist view that everything social is just the actions of individual people, and the post-modernist, holist view that psychological reality is a matter of social construction. We will show that our part-whole schema provides an alternative answer to the people/group problem by considering a particularly important kind of social group—families.

All human cultures have families (Brown, 1991), making them the most pervasive kind of social group. A family is obviously a whole whose parts are the people who belong to it, but what are the tags, organizers, attachers, and communicators? To simplify the analysis, we will consider the typical Western family consisting of two parents and a few children, but it would be straightforward to broaden the account to the more diverse, extended families found in various cultures.

Tags are properties of parts that provide identities enabling the parts to be assembled and maintained as wholes. The properties of people that enable them to belong to families are physiological, behavioral, psychological, and neuromolecular. Physiological differences enable people to recognize individuals. People need behaviors such as talking and touching to function as members of families, but these behaviors result from psychological mechanisms, which in turn depend on underlying neural mechanisms. The psychological mechanisms, according to research in cognitive science, are computational processes that operate on many kinds of mental representations including concepts, beliefs, goals, attitudes, and emotions (see e.g., Thagard, 2005; Smith and Kosslyn, 2007). All of these can contribute to the identifications that form people into families.

The concepts that people need to function as parts of a typical Western family include *wife, husband, father, son, daughter*, and *family* itself. Categorizing oneself as a parent or child provides a way of thinking of oneself in relation to the family. These categories are tied to emotional attitudes about the roles that people play in the family, and family stability depends on the existence of emotional bonds deriving from positive attitudes about being a spouse or a child. These cognitive/emotional mechanisms depend on biological processes that are both neural and molecular. For example, coupling depends on molecular processes involving neurotransmitters such as dopamine (for positive attitudes) and hormones such as oxytocin (for bonding).

Merely having these tags, however, does not suffice to establish and maintain groups like families. From the perspective of our part-whole schema, formation of families requires organizers, which are forces and processes that bring parts together into relationships. The organizers for families in Western societies are multiple and diverse, including at least the following. The forces behind family formation include sexual desire, need for relatedness or belonging, and motivation to satisfy social expectations. The processes by which people form into couples include social practices such as schooling, work, parties, dancing, and dating. Marriage rituals, whether religious or secular, provide public occasions for social recognition of the formation of a family. The expansion of a family by addition of children requires both psychological processes, such as the desire to reproduce or at least to have sex, and social processes such as assistance in childbirth by medical personnel and hospitals. Adoption is another social process by which a family can be expanded.

Note that whereas the tags behind families are largely the psychological properties of individuals, the organizers for families include social processes as well as psychological ones. Hence it should be clear that the account of families based on our part-whole schema does not attempt to reduce families to their individual members, because social processes such as schooling, rituals, and healthcare are crucial to the organizing of families.

In our part-whole schema, attachers are forces, processes, or entities that hold parts together, and the maintenance of families depends on multiple attachers. Some of these are psychological and neural, such as the representational and neurochemical processes that underlie people staying in love, whose neural mechanisms include activity in brain reasons rich in dopamine (Fisher, 2004). Other processes are social, such as the legal status of marriage which makes dissolution of families non-trivial. For young children, physical and psychological dependency are powerful attachers that naturally wane with maturity. Despite the independence of grown children, family identity can be maintained through ongoing social interactions such as family dinners and other visits, including ones associated with family or cultural rituals.

These connections depend on communicators that allow interactions among physically separated parts. Besides speech, communication between family members can take place through music and many kinds of technology, including mail, telephones, e-mail, and Internet-based chat. Such communication depends on individual tags that enable people to generate and receive messages, including the psychological processes required for language production and comprehension.

Families illustrate how constitution of parts into wholes requires the whole complex of tags, organizers, attachers, and communicators. The tags of the parts allow them to be organized by forces and processes and attached together into wholes in which communicators provide additional interactions among parts. Consider the property of being a *happy family*, which has both an aggregate and an emergent interpretation. As an aggregate property, a happy family is just one in which all the individuals are happy, but there is also a more interesting interpretation in which the happiness of the family results from ongoing interactions between individuals based on processes of organizing, attaching, and communicating. The happiness of the family as a whole depends on how its members treat each other. Similarly, a dysfunctional family need not be one in which each of the individuals is dysfunctional; rather, the interrelations among the individuals render the family dysfunctional. Family conflict is obviously not a property of individuals, but an ongoing process depending on how individuals interact.

We have given only a cursory account of how individuals function in families, but it suffices to show the applicability of our part-whole schema to a fundamental kind of social group. Expanded accounts could be given of many other kinds of social organization, including institutions and even nations. Our answer to the people/group problem is that people constitute groups by virtue of a large array of tags, organizers, attachers, and communicators. Many of these factors are neuropsychological, but social interactions are also a crucial part of the story of how families consist of people. Mental representations such as concepts and emotions are needed to explain the formation and maintenance of groups, but also highly relevant are social practices that depend both on the representations that people have of each other and on the various kinds of interactions that bring and keep people together. We hope it is clear that our account is neither simplistically reductionist nor mystically holistic, but rather shows that the part-whole schema supports a rich, multilevel account of the relation between people and groups.

## Conclusion

Philosophers have debated the nature of parts and wholes since Plato (Harte, 2002; Wasserman, 2009). Our account is broadly compatible with many recent discussions of parts and wholes in relation to mechanisms and levels in recent philosophy of science (e.g., Bechtel and Richardson, 1993; Bunge, 2003; Bechtel, 2006, 2008; Darden, 2006; Craver, 2007; Craver and Bechtel, 2007; Wimsatt, 2007; Winther, 2011). However, it goes beyond these discussions in providing a more detailed account that operates at all scientific levels, from the atomic to the social, and in specifying the properties of parts (tags) that allow them to be organized into wholes and maintained as wholes by processes of attachment and communication. These specifications have enabled us to describe how part-whole relations operate in an important kind of social structure, the family.

Our science-based part-whole scheme is very different from the conceptual, *a priori* accounts of parts and whole offered in analytic metaphysics (e.g., Simons, 1987; Sider, 2007; Varzi, 2009; Schaffer, 2010). Such accounts lead to broad claims incompatible with scientific discourse, such as that wholes are prior to parts and that everything is a part of itself. In contrast, we have attempted to characterize how parts make up wholes by looking at examples from a broad range of sciences.

The resulting part-whole schema shows how constitution is established and maintained at different levels, in ways that are dynamically dependent on ongoing causal interactions. Instead of taking constitution as primitive, we have provided a scientifically realistic account of the structures and processes that make parts into wholes. This account has served to illuminate the nature of social structures such as families, demonstrating the commonality of part-whole relations from the atomic level to the biological and social.

### Conflict of interest statement

The authors declare that the research was conducted in the absence of any commercial or financial relationships that could be construed as a potential conflict of interest.
